# Culturally Adapting the World Health Organization Digital Intervention for Family Caregivers of People With Dementia (iSupport): Community-Based Participatory Approach

**DOI:** 10.2196/46941

**Published:** 2024-01-24

**Authors:** Anna Messina, Rebecca Amati, Anna Maria Annoni, Beatrice Bano, Emiliano Albanese, Maddalena Fiordelli

**Affiliations:** 1 Institute of Public Health Faculty of Biomedical Sciences Università della Svizzera italiana Lugano Switzerland

**Keywords:** informal caregivers, iSupport, dementia, digital interventions, mHealth, community-based participatory research, community, caregiver, mental distress, physical distress, support, development

## Abstract

**Background:**

Informal caregivers of people with dementia are at high risk of developing mental and physical distress because of the intensity of the care provided. iSupport is an evidence-based digital program developed by the World Health Organization to provide education and support for the informal everyday care of people living with dementia.

**Objective:**

Our study aims to describe in detail the cultural adaptation process of iSupport in Switzerland. We specifically focused on the participatory strategies we used to design a culturally adapted, Swiss version of iSupport that informed the development of the desktop version, mobile app, and printed manual.

**Methods:**

We used a mixed methods design, with a community-based participatory approach. The adaptation of iSupport followed the World Health Organization adaptation guidelines and was developed in 4 phases: content translation, linguistic and cultural revision by the members of the community advisory board, validation with formal and informal caregivers, and refinement and final adaptation.

**Results:**

The findings from each phase showed and consolidated the adjustments needed for a culturally adapted, Swiss version of iSupport. We collected feedback and implemented changes related to the following areas: language register and expressions (eg, from “lesson” to “chapter” and from “suffering from” dementia to “affected by” dementia), resources (hyperlinks to local resources for dementia), contents (eg, from general nonfamiliar scenarios to local and verisimilar examples), graphics (eg, from generalized illustrations of objects to human illustrations), and extra features (eg, a glossary, a forum session, and a read-aloud option, as well as a navigation survey).

**Conclusions:**

Our study provides evidence on how to culturally adapt a digital program for informal caregivers of people living with dementia. Our results suggest that adopting a community-based participatory approach and collecting lived experiences from the final users and stakeholders is crucial to meet local needs and to inform the further development, testing, and implementation of digital interventions in a specific cultural context.

## Introduction

### Background

Approximately 55 million people are currently living with dementia worldwide [1]. Switzerland accounts for >150,000 cases of dementia, with an expected doubling by 2050 [2]. In Switzerland, as in most countries, the majority of people living with dementia live at home assisted by an informal caregiver, who is usually a family member who provides daily support and coordinates care delivery [3]. There are positive outcomes that may be associated with the caring role, such as the perception of a better relationship and closeness with the care recipient [4]. Nonetheless, the increasing complexity of taking care of a person affected by dementia exposes informal caregivers to psychological distress and increases the risk of loneliness and developing symptoms of anxiety and depression [5,6]. Caregivers’ psychological distress is also associated with a lower quality of care provided [7] and with the worsening of behavioral and psychological symptoms in the care recipients [8].

Providing guidance and support to informal caregivers is one of the priority areas identified by the World Health Organization (WHO) to reduce the global impact of dementia and to improve the quality of life of caregivers and their families [9]. In the last 2 decades, digital educational and psychosocial interventions for caregivers have bloomed [10,11]. Internet-based interventions are more easily accessible [12,13] and adaptable to the time and geographic constraints of caregivers [14]. Some reviews suggest that multiple components of digital interventions can contribute to reducing the burden and improving the quality of care and be even more beneficial if tailored to caregivers’ specific needs and contexts [10,15,16]. The active involvement of the final users and relevant stakeholders in the design and local adaptation as well as the testing and piloting of interventions is crucial for need-centered interventions in terms of their uptake, integration, and scalability at the community level [17,18]. However, more evidence is required to understand the most effective methods and strategies needed to involve participants in the design and adaptation of digital interventions [19,20].

**Figure 1:**
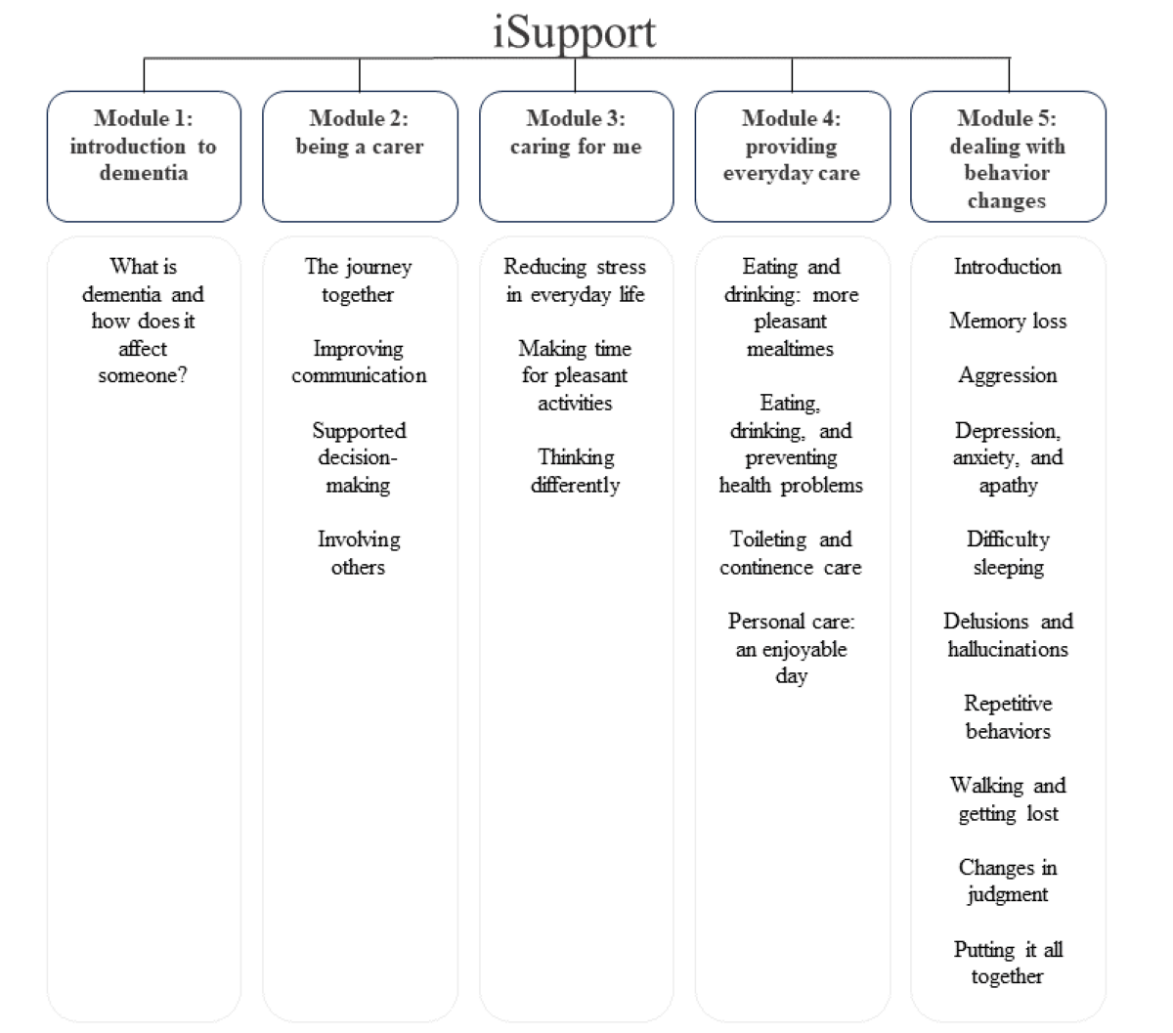
iSupport index.

iSupport is an evidence-based digital training intervention developed by the WHO to provide support and education to informal caregivers of people with dementia [21]. The original program consists of 22 thematic lessons distributed across 5 modules (Figure 1). Each lesson covers a specific topic associated with care that ranges from the daily assistance of the care recipient (eg, toileting, personal care, and nutrition) to the self-care of the carer (eg, reducing stress and involving others in care duties). All lessons include theoretical and informative sections and case scenarios with interactive multiple-choice questions. The WHO provides guidelines to culturally adapt iSupport contents to the local language, culture, and context before implementation [22]. The reporting of adaptation processes of complex interventions is limited but extremely important [23]. Knowledge exchange of methodologies and approaches as well as evidence on barriers and facilitators to local adaptation are crucial preliminary steps to inform the implementation of interventions and their mid- to long-term uptake and sustainability [24].

### Objectives

This study aims to describe in detail the cultural adaptation process of iSupport in Switzerland. We specifically focused on the participatory strategies we used to design a culturally adapted, Swiss version of iSupport that informed the development of the desktop version, mobile app, and printed manual. Our purpose is to inform the implementation of not only iSupport but also other complex health interventions, specifically in the context of the cultural adaptation process.

## Methods

### Study Setting

The study took place in the Italian-speaking part of southern Switzerland, namely the canton of Ticino.

### Ethical Considerations

Before initiating the study, we sought ethics oversight by submitting our project to the cantonal ethics committee, and we obtained a waiver of ethics approval and official authorization to proceed with the study (project ID: 2020-02030 / CE 3731). Specifically, the ethics committee determined that our project did not fall within the scope of the Swiss Federal Human Research Act [25], thereby granting us permission to proceed.

### Theoretical Approach

The overall process of culturally adapting iSupport in Switzerland was based on principles from the community-based participatory research (CBPR) framework, which can be defined as “an approach to research that involves collective, reflective and systematic inquiry in which researchers and community stakeholders engage as equal partners in all steps of the research process” [26]. In intervention research, adopting CBPR has the advantage of facilitating knowledge exchange between the community and the researchers, reducing potential power imbalances, and increasing the likelihood of intervention uptake and success [27].

We based the specific phases and procedures of the adaptation process on the WHO iSupport adaptation guidelines [22], which, in turn, are based on the ecological validity framework proposed by Bernal et al [28] that is widely used for developing culturally sensitive interventions and strengthening their ecological validity [29-31].

### Study Procedure

#### The Community Advisory Board

At the outset, we established a community advisory board (CAB) comprising community members and organization representatives who shared a common identity, geography, language, culture, and other values and principles [32].

We identified potential members of the CAB through a structured stakeholder analysis and mapping that accounted for the different levels of power, importance, and interest of the stakeholders in the project. We included representatives of the project’s funding agencies and other collaborating partners, caregivers of people with dementia, and members of the IT service in charge of developing the iSupport web platform and app. Once consensus among researchers was reached, we contacted and informed the identified members via email using a brief description of the project, the scope of the CAB, and their expected roles and responsibilities.

In the context of iSupport adaptation, the specific roles of establishing a CAB were to (1) help researchers to identify the needs and legitimate interests as well as the expectations of the different stakeholders and the final users and (2) inform the development of the intervention throughout a purposely co-designed process.

**Figure 2:**
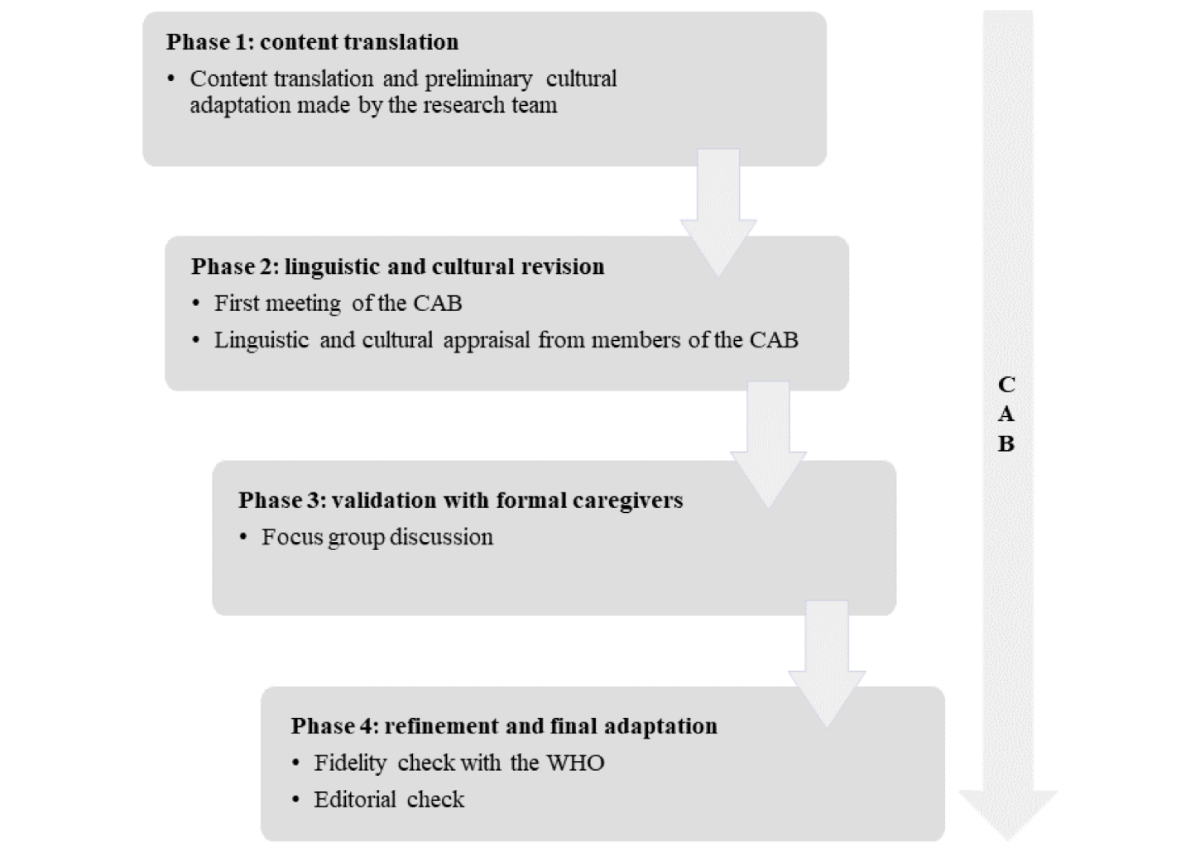
Flowchart of the adaptation process of iSupport in Switzerland. CAB: community advisory board; WHO: World Health Organization.

The adaptation process of iSupport consisted of four phases: (1) content translation, (2) linguistic and cultural revision, (3) validation with formal and informal caregivers, and (4) refinement and final adaptation. Each phase was based on, and adapted from, the WHO guidelines. Any change or proposed addition was discussed with, and approved by, the WHO. The members of the CAB were constantly informed and updated on the progress of the study. A flowchart of the phases is summarized in Figure 2.

#### Phase 1: Content Translation

The first step in the cultural adaptation of iSupport was the translation of the contents (approximately 60,000 words) from English, the original language of the program, into Italian, the local language in southern Switzerland. The process started in May 2020 and ended in August 2020. According to the WHO guidelines, the translation should be accurate while recognizing the local culture and its people. In line with this, we conducted a preliminary adaptation of culturally sensitive terms, including (1) personal names of the characters used in the case studies, (2) available information materials and local services, and (3) reference to cultural habits and leisure activities in the region [22].

One member of the research team fluent in English, AM, a psychologist with previous expertise in the dementia field, translated the original contents of the iSupport program into Italian. Subsequently, a senior member of the team, MF, with expertise in the field of health communication, checked the translations and proposed changes and modifications. All disagreements or doubts about the translation of sensitive terms and expressions were documented and discussed within the research team in meetings until a consensus was reached. We sought the support of an external professional translator to resolve some specific language locutions and terms.

Throughout the process, translators applied the international standards and available dementia guidelines to avoid stigmatizing expressions and to use language that promotes the inclusion and dignity of people living with dementia and their carers [33]. During this phase, we did not apply any changes to the meanings of the original structure of the iSupport program, including case studies or activities. All translations were copied into secure Microsoft Word files and stored in a dedicated Microsoft Teams workspace to optimize efficiency.

#### Phase 2: Linguistic and Cultural Revision

In September 2020, the first CAB meeting took place with the main goals of introducing the members of the CAB to the iSupport program and the research team and clarifying their roles and involvement throughout the research process. During the meeting, we answered all questions and proposed an interactive activity where participants were asked to provide the translation from English into Italian of a selection of sensitive terms and expressions used in iSupport that were noted by researchers during phase 1. At the end of the introductory meeting, participants were asked to sign a letter of intent that summarized the functioning of the CAB and their role and commitment as members of the local iSupport CAB. We explicitly specified the structure and definition of the CAB; goals, roles, and responsibilities (of both CAB members and the research team); and duration (Multimedia Appendix 1). All 9 invited participants agreed to join the iSupport CAB: 4 (44%) were informal caregivers, and the remaining 5 (56%) included representatives of the government (1/5, 20%), the local Alzheimer association (1/5, 20%), a health care service provider (1/5, 20%), the IT service (1/5, 20%), and the University of Applied Sciences and Arts of Southern Switzerland (1/5, 20%).

In October 2020, we shared the translated contents of iSupport with the members of the CAB and asked them to evaluate, and provide feedback on, each chapter and module of the program by the end of December 2020.

On the basis of the work of previous adaptations of iSupport [34], participants were asked to carefully go through the 23 thematic lessons and assess the translation and preliminary adaptation of iSupport considering six main parameters: (1) familiarity, (2) sensitivity, (3) comprehensibility, (4) precision, (5) cultural adequacy, and (6) overall evaluation. In addition, they were asked to assess the extent to which (1) the terms used were familiar to the target group (eg, the use of idioms and figures of speech), (2) the language used respected and promoted the dignity of people living with dementia and their carers (eg, the use of stigmatizing terms), (3) the contents were intelligible and easy to understand (eg, minimal use of technical jargon), (4) the contents were presented in an accurate way (eg, they were in accordance with the facts, and there were no mistakes), (5) the contents were appropriate and reflected the experiences of local people (eg, case studies), and (6) the content of each chapter was overall culturally appropriate. At the end of each chapter, participants were asked to fill out a digital survey via Research Electronic Data Capture (REDCap; Vanderbilt University) [35,36] to evaluate each of the aforementioned parameters using a Likert scale ranging from 1=*requiring an extensive revision* to 4=*no additional revision needed*. We also invited participants to provide additional comments about individual chapters through a dedicated open-ended question in the survey or to provide free feedback on the overall program via email. The survey was specifically designed for the purpose of this phase and was based on the work of Teles et al [34] to evaluate the cultural adequacy of the contents, as recommended by the WHO adaptation guidelines [22].

After the data collection period, AM and BB (a research assistant with a degree in psychology and health communication) performed a descriptive analysis of the quantitative data and a thematic analysis of the qualitative data. For the quantitative analysis, we used SPSS statistical software (version 25.0; IBM Corp) [37] for Windows to compute mean scores for each program module and survey parameters. For the qualitative analysis, we performed a thematic content analysis of open comments [38]. The maintenance of scientific rigor was ensured through regular meetings among research team members, particularly involving MF and RA, both experts in qualitative research methods.

In January 2021, the main findings of this phase were summarized in a report shared across, and approved by, all CAB members.

#### Phase 3: Validation With Formal and Informal Caregivers

We adopted a qualitative descriptive design, and we used focus groups (FGs) as a data collection method [39]. Between June 2021 and August 2021, we conducted FGs with formal and informal caregivers to explore their attitudes toward, and impressions of, the adapted version of iSupport. We prompted and collected suggestions for improvement, as also recommended by the WHO guidelines. In addition, we decided to expand our inquiry to caregivers’ attitudes toward support measures and help-seeking behaviors, which we have previously reported in detail elsewhere [40].

From April to May 2021, we crafted an invitation letter and a flyer presenting the project, the main purpose of the FGs, the eligibility criteria, and contact information. We disseminated these materials in a local newspaper, to members of the CAB and their associations and institutions, to daycare centers for people with dementia, and to participants of other ongoing research projects who had consented to be informed about further research activities.

Eligibility criteria for both formal and informal caregivers included (1) having (at present or in the past) experience in caring for a person living with dementia, (2) being fluent in Italian, and (3) living in the canton of Ticino. Caregivers who met the inclusion criteria could contact us via email or telephone. Those who contacted us were given an overview of the iSupport program, with excerpts from the same translated material used in phase 2, and the informed consent form (Multimedia Appendix 2). The FGs, which lasted approximately 2 hours, were audio recorded and took place either digitally via the Zoom platform (Zoom Video Communications, Inc) or in person at the Università della Svizzera italiana in Lugano, Ticino. AM moderated all FGs, with the supervision of MF and RA. The discussions were transcribed verbatim and pseudonymized by EB, an independent research assistant. AM, RA, and MF performed a thematic content analysis to identify key themes [38]. Initially, the researchers familiarized themselves with the data through repeated reading of notes and transcripts to get an idea of the overall meaning and begin discerning key themes. Subsequently, each researcher independently identiﬁed codes within each FG (vertical analysis) and across the whole data set (horizontal analysis) to uncover variations and patterns within the data. Themes were progressively refined and consolidated through discussion in weekly meetings over 4 months (from November 2021 to February 2022) and until a consensus was reached. Data management and coding processing were facilitated by NVivo 12 software (Lumivero) [41]. Additional methodological details have been previously reported [40].

#### Phase 4: Refinement and Final Adaptation

All data collected during phases 2 and 3 were collated to generate a set of proposed changes and adaptations to the iSupport program. AM and BB familiarized themselves with the data and differentiated the feedback between cross-cutting and general comments and specific chapter–related comments and arranged them across 5 dimensions: language, resources, contents, graphics, and extra features. Each comment was then discussed between AM and BB and categorized as (1) *rejected/not applicable*, (2) *possibly applicable*, and (3) *applicable*. The categorization was based on the number and contents of suggestions received as well as in accordance with the WHO adaptation guidelines [22]. The feedback data that were considered *possibly applicable* and *applicable* were then discussed with the other members of the team to agree on their potential implementation.

Subsequently, all specific and applicable comments were charted using the iSupport WHO adaptation spreadsheet (Multimedia Appendix 3). All proposals of change were then supported by a rationale and by the source of the proposal: the research team (preliminary adaptation during phase 1), the members of the CAB (results from the linguistic and cultural adaptation during phase 2), and FG participants (data collected during phase 3). Attached to the adaptation spreadsheets, we also sent a list of general suggestions and feedback. The material was sent for revision to the authors of the WHO iSupport program in October 2021, and the results of their final fidelity check were received in January 2022. Subsequently, the local research team implemented all approved changes and uploaded the new adapted contents on the beta version of the iSupport Swiss web platform.

## Results

A detailed description of all final adaptations made to the original iSupport program, resulting from the 4 phases of the adaptation process, is presented in Multimedia Appendix 4. The results of phases 2 and 3 are summarized in the subsections that follow.

### Adaptations From Phase 2: Linguistic and Cultural Revision

#### Overview

All 9 members of the iSupport CAB revised ≥1 modules of iSupport and provided feedback, with each module revised by at least 1 CAB member. Module 5, which dealt with behavior changes, was the most revised and received the most comments (7/9, 78%). Descriptive analysis showed that all modules were generally positively evaluated with an overall mean evaluation score of 3.72 (SD 0.00) out of 4. Mean scores of the parameters across all modules ranged from 3 (SD 0.00) to 3.89 (SD 0.47) for sensitivity, from 3 (SD 0.00) to 4 (SD 0.00) for familiarity, from 3.93 (SD 0.26) to 4 (SD 0.00) for comprehensibility, from 3.67 (SD 0.58) to 4 (SD 0.00) for the accuracy of the information, and from 3.59 (SD 0.62) to 4 (SD 0.00) for cultural adequacy (Table 1).

We conducted a qualitative thematic analysis of the open comments and identified 7 potential areas for the improvement of iSupport (for more details, refer to Multimedia Appendix 4).

**Table 1:** Mean scores of the 6 parameters for the linguistic and cultural revision of iSupport.

Parameter	Module 1, mean (SD)	Module 2, mean (SD)	Module 3, mean (SD)	Module 4, mean (SD)	Module 5, mean (SD)
Sensitivity	3.60 (0.55)	3.59 (0.62)	3.00 (0.00)	3.87 (0.52)	3.89 (0.47)
Familiarity	3.60 (0.55)	3.65 (0.61)	3.00 (0.00)	3.67 (0.62)	4.00 (0.00)
Comprehensibility	4.00 (0.00)	3.94 (0.24)	4.00 (0.00)	3.93 (0.26)	4.00 (0.00)
Accuracy	3.75 (0.50)	3.76 (0.44)	3.67 (0.58)	3.73 (0.70)	4.00 (0.00)
Cultural adequacy	3.60 (0.55)	3.59 (0.62)	4.00 (0.00)	3.80 (0.56)	3.89 (0.47)
Overall assessment	3.20 (0.45)	3.65 (0.49)	3.00 (0.00)	3.80 (0.41)	4.00 (0.00)

#### Familiarity With Terms and Expressions

The unfamiliarity with the terms referred especially to some expressions that were largely used throughout the text to designate caregivers and health care workers or dementia health and social care facilities, and the comments by the CAB members allowed us to improve the translations; for instance, “informal carers” and “paid in-home helpers” were newly translated using local terms that were easily identifiable and familiar to participants (eg, “informal carer” was replaced with “familiare curante,” which literally means “family carer”). Importantly, the term is also used at an institutional level [42] to refer to people who take care of a loved one (relative or friend) affected by a chronic disease.

#### Sensitivity of the Language

Participants also suggested improving the sensitivity of the language by removing expressions potentially stigmatizing such as “suffering from dementia,” which was replaced with “affected by dementia” (*affetto da demenza*), or “show compassion,” which was replaced with “show comprehension” (*mostrare comprensione*).

#### Scientific Accuracy of the Terms

The jargon used was generally perceived as comprehensible and easy to understand. However, some of the participants (2/9, 22%), especially those working in the field of dementia, reported the need to use scientific terms to improve the accuracy of the language and ultimately the users’ literacy. As a result, the expression “memory loss” was, for example, replaced with “memory impairment” (*difficoltà di memoria*), and “helpful/unhelpful thoughts” was replaced with “functional or dysfunctional thoughts” (*pensieri funzionali e disfunzionali*).

#### Educational Approach

The educational approach referred to the use of terms considered scholastic and potentially belittling by participants, such as “lesson” and “learn,” which were replaced with “chapter” (*capitolo*) and “know more about” (*conoscere di più*), respectively.

#### Use of English

Some English terms that were retained during the content translation because they are normally used in spoken Italian were translated into Italian, including “focus” (*obiettivo*) and “relax” (*rilassa*).

#### Use of Numbers

To make the reading smoother, some of the participants (3/9, 30%) suggested replacing numbers with sentences (eg, from “20%-30%” to “approximately one-third”; *circa un terzo*).

#### Language Register

Finally, almost all participants (8/9, 89%) found that the tone and prose were at times informal or even childish; therefore, for example, the original sentence at the end of each chapter “you finished the lesson, well done” was replaced with “you finished the chapter, let’s go to the next!” (*hai completato il capitolo, passa al successivo!*).

### Adaptation From Phase 3: Validation With Formal and Informal Caregivers

#### Overview

Between May 2021 and August 2021, we conducted 6 FGs: 1 (17%) with formal caregivers and 5 (83%) with informal caregivers. Most of the participants (16/19, 84%) were female, and the formal caregivers (6/19, 32%) had longer years of caring experience than the informal caregivers (13/19, 68%). The main characteristics of the caregivers are reported in detail in Tables 2 and 3.

**Table 2:** Sociodemographic characteristics of formal caregivers.

ID	Sex	Age (y)	Employment status	Years of professional caring experience	Years of personal caring experience
1	Female	52	Housewife or retired	>10	>10
2	Female	54	Housewife or retired	>10	>10
3	Female	28	Housewife or retired	6-10	0
4	Female	45	Employed	<1	<1
5	Female	29	Housewife or retired	1-2	0
6	Female	59	Employed	>10	0

**Table 3:** Sociodemographic characteristics of informal caregivers.

ID	Sex	Age (years)	Employment status	Relationship with the person with dementia	Living situation of the person with dementia	Years of caring experience	The person with dementia has passed away	Focus group attended^a^
1	Female	58	Housewife or retired	Spouse	Own residence	3-5	No	5
2	Female	55	Housewife or retired	Daughter	Own residence	3-5	No	1, 3, and 4
3	Female	59	Housewife or retired	Spouse	Own residence	3-5	No	1
4	Male	67	Employed	Son	Own residence	3-5	No	1
5	Female	58	Housewife or retired	Spouse	Carer’s residence	1-2	No	2
6	Male	57	Employed	Son	Own residence	3-5	No	1 and 4
7	Male	74	Employed	Son	N/A^b^	>10	Yes	1 and 2
8	Female	55	Employed	Daughter	Own residence	3-5	No	2 and 4
9	Female	75	Housewife or retired	Daughter	N/A	>10	Yes	1, 2, 4, and 5
10	Female	76	Housewife or retired	Spouse	Own residence	3-5	No	2
11	Female	82	Housewife or retired	Spouse	Carer’s residence	1-2	No	2
12	Female	55	Employed	Daughter	Own residence	1-2	No	3 and 4
13	Female	81	Housewife or retired	Spouse	Carer’s residence	3-5	No	2 and 4

^a^Number of the focus group attended.

^b^N/A: not applicable.

#### FG With Formal Caregivers

Formal caregivers are professionals who are trained, hired, and paid to provide care to a person living with dementia. In our study, all formal caregivers (n=6) actively participated in the digital discussions. All participants were female. Their mean age was 44 (range 28-59) years. All participants had professional experience in taking care of a person living with dementia. Of the 6 participants, 3 (50%) reported having >10 years of experience in dementia care. In addition to the professional caregiving experience, 3 (50%) of the 6 participants also reported taking care, or having taken care in the past, of a relative affected by dementia (Table 2). The main findings of the FG are summarized in the following paragraphs.

Participants agreed that an intervention aiming to support and improve the knowledge of informal caregivers of people with dementia was much needed. A caregiver compared information learning to a safeguard not only for the carer but also for the care recipient:

I hope that this program will spread because information protects all of us: the carer, and especially the person who is cared for.

ID 6

iSupport was generally appreciated and acknowledged by participants as a useful tool. The contents were found appropriate and sufficiently comprehensive. The difficulty regarding accepting the disease and the changes in the relationship with the care recipient were found to be the main challenges and contents to cover in the program:

Relatives find it extremely difficult to accept the disease and the change...I believe a very strong support is needed...also at a social level because the disease is often associated with shame.

ID 1

Similarly, a participant also suggested adding to the program specific resources for social and psychological support:

You could mention [the existence or the opportunity for family members] to benefit from psychological support because they need it, always.

ID 2

This quote underscored the recognition by formal caregivers of the potential emotional and psychological strain on family members as they witness the progression of the disease of their care recipients.

In light of the participants’ perspectives, an important feature to add to the original iSupport format was the inclusion of a platform for caregivers to engage with each other and that facilitated the caregivers’ interactions with each other (this adaptation was also needed to differentiate iSupport from another repository of information or digital available resources on dementia):

There are a billion guides on dementia...I think people need to interact.

ID 4

Regarding case scenarios, the caregivers generally found that the examples were appropriate and consistent with their experiences. However, the answer options often did not reflect the variety of, and differences in, caregiving situations and experiences, including the age of the person affected by dementia, the severity of dementia, the living situation, or the type of dementia (eg, Alzheimer disease and frontotemporal dementia). A participant suggested adding general guidelines to the examples to include more answers:

If the examples aim to increase knowledge, they should give general indications that can apply to different caring situations.

ID 6

#### FG With Informal Caregivers

Of the 20 informal caregivers who contacted us, 13 (65%) joined the FGs. Reasons for nonparticipation were lack of time and geographic distance. Most of the caregivers (10/13, 77%) were female; nearly half were spouses (6/13, 46%) of the persons living with dementia, and more than half were children (7/13, 54%; daughter: n=4, 57%; son: n=3, 43%) of the persons living with dementia. Their age ranged from 55 to 82 years. Most of the participants (10/13, 77%) reported a caregiving experience of at least 3 years, and most of them (8/13, 62%) cared for a relative who lived at their own residence. Of the 13 participants, 2 (15%) reported that the person they cared for had passed away. The number of caregivers attending each FG ranged from 2 to 7: of the 13 caregivers, 6 (46%) attended FG 1 on June 14, 2021; a total of 7 (54%) attended FG 2 on July 12, 2021; a total of 2 (15%) attended FG 3 on July 15, 2021; a total of 6 (46%) attended FG 4 on August 18, 2021; and 2 (15%) attended FG 5 on August 24, 2021. Of the 13 participants, 7 (54%) attended >1 FG (Table 3). The main findings of the 5 FGs are summarized in the following paragraphs.

Participants generally believed that iSupport holds the promise to be useful, to increase dementia knowledge, and provide information about available services and support measures for people living with dementia and their families:

The idea is brilliant because everything can be useful...In my opinion, the most interesting thing is the overview of what is locally available to support caregivers.

ID 9; daughter

The need for guidance and orientation to services was felt owing to a perceived lack of support and direction, likely stemming from the uncertainty and confusion that frequently followed the diagnosis. This feeling of bewilderment was echoed by a participant:

It’s confusing outside, you don’t know where to go, whom to turn to...there are no guidelines, no support.

ID 2; daughter

Regarding the contents, participants reported familiarity with most of the case scenarios.

A participant commented as follows on a scenario (module 3, chapter 3) involving a person affected by dementia who cannot find the house keys and does not want the carer to leave him alone at home:

It happened to me many times, not always with the keys though.

ID 12; daughter

However, despite the familiarity reported and the need to obtain information and increase knowledge to cope with difficult situations, the original exercise format was seen as a limitation by some of the participants. A participant reported feeling diminished when choosing between wrong and right answers:

It’s almost guilt-inducing...There is the best solution and if you guess wrong you are doing your role wrong.

ID 8; daughter

In addition, some of the answer options were considered to be so wrong as to be offensive to the carer; for example, in module 5, chapter 9, a case scenario described a situation where the person affected by dementia (Matteo) makes sexual remarks toward a domestic worker, and the user is asked what they would do in this situation. A participant commented on the option “shout at Matteo and shame him for his conduct” as inconceivable:

Shout?!...We do know what we’re doing!

ID 9; daughter

Similar to what formal caregivers reported about case scenarios, participants also highlighted the risk of generalizing solutions that may not be appropriate for all caregiving situations:

It should be clear that each user has to transpose his or her situation by taking cues from the scenario, but unfortunately it isn’t black and white.

ID 13; spouse

Finally, the informal caregivers too suggested adding interactive features to the digital version of iSupport to minimize the risk of the caregivers isolating themselves; for instance, a participant commented as follows:

For me, the biggest utility is in connecting people...there should be people behind the app.

ID 5; spouse

## Discussion

### Overview

This study described in detail the main steps taken to culturally adapt the WHO iSupport program for informal caregivers of people living with dementia in Switzerland. Our results demonstrate the complexity as well as the necessity of adapting an evidence-based complex intervention to a specific cultural context and population. We collected feedback and implemented changes, in accordance with the WHO authors of the program, to the original iSupport version in the areas of the language, resources, contents, graphics, and features used in the program. In the following paragraphs, we summarize and comment on the main lessons learned.

### Valuing Experiential Knowledge

One of the main messages we took away during the adaptation process was the importance placed by informal caregivers on being recognized for their role and expertise. This finding is consistent with the findings of other studies, including the works reporting on iSupport adaptation processes in other countries [31,34,43-45]. Our participants suggested that the learning approach used in the original iSupport program was too scholastic and recommended the removal of expressions that likely resulted from a top-down approach to content and compilation. Referring to case scenarios, some of the informal caregivers (5/13, 38%) felt that the simplicity of certain answer options was offensive. Informal caregivers claimed to be recognized because of their lived experience as *experts in the field* who could contribute to not only locally adapting iSupport but also integrating and shaping it. This echoes the inclusive procedures used to develop iSupport in the first place [21] and the work done for the iSupport adaptation process in Portugal and the United Kingdom [34,43]. Informal caregivers can spend on average 170 hours a month providing care to a loved one affected by dementia [46]. In our study, more than half of the caregivers (10/13, 77%) reported a caregiver experience of at least 3 years and up to 10 years. Although one may argue that caregivers acquire and improve their learning by doing, it is undeniable that they can become *experts* in caring; surely, they provide a unique perspective of the person with dementia and their own needs. However, besides the years of personal experience, caregivers’ knowledge of dementia and caring may also depend on other factors and may be influenced by their educational level and sociocultural background. Similar to any complex health intervention [47], it is important to ensure that the final version of iSupport is adapted to the real user’s experience and preexisting abilities. An early, timely, and active involvement of caregivers is needed [48,49]. The adoption of a language register and skills training techniques that promote preexisting abilities, rather than replace them, may enhance the acceptance and use of the intervention.

### Enhancing Social Contacts

According to participants, iSupport could benefit from the inclusion of interactive features (eg, chat and forum) that allow the user to communicate with other caregivers and share experiences and problem-solving strategies. This finding is consistent with a recent study [50] that found that peer support can be complementary to professional support and beneficial in reducing social isolation, as well as in connecting patients and caregivers to others with similar issues. Similarly, Greenwood et al [51] found that, besides providing psychosocial support, peer support interactions for caregivers of people with dementia can offer practical information and guidance in managing difficult situations and gaining new perspectives on their caring role.

The adoption of peer support programs for informal caregivers of people with chronic diseases and disabilities is well established in the literature [52]. A recent scoping review [53] found that peer support was often part of multicomponent interventions that also addressed information sharing, skills development, personal coping skills, and self-management. Despite the difficulty in identifying what component may or may not be beneficial for the carers, the authors concluded that peer support, particularly if delivered digitally, could represent a cost-effective medium and opportunity to meet caregivers’ needs and preferences.

Importantly, digital meets among peers seem more promising, usable, and potentially effective for caregivers when embedded in digital interventions [10] such as iSupport.

### Facilitating Access to, and Navigation of Local Services

Another suggested feature to implement in the program was the inclusion of contacts of local resources for dementia, such as health care services and facilities, charities, or other relevant organizations. Consistent with what our participants reported, informal caregivers often experience a lack of information and support, especially at the beginning of the caregiver journey, when it is best to establish fruitful contacts and interactions with local health and social care services and offers in general [1]. According to the latest World Alzheimer Report [3], <50% of informal caregivers are advised to contact the local Alzheimer association or receive postdiagnostic support information. The navigation of the services and various offers for both people living with dementia and informal caregivers is taxing, often ineffective, and can be frustrating. The lack of information about existing services and support is associated with caregiver burden and distress [54]. A recent review on the needs of family caregivers revealed that information provided on available support services and measures was one of the main needs reported by caregivers after their loved one was diagnosed with dementia [55]. Caregivers may seek support autonomously, mainly digitally. However, the variety of information and sources available on the internet about dementia may contribute to creating feelings of bewilderment and difficulties in finding relevant and reliable information [56]. Hence, digital interventions that also include contacts with external and local resources may help users to access and navigate the health care system and find the most appropriate service or information for their situation.

### Limitations

We acknowledge that our study has limitations. First, we included only a few participants for each phase of the adaptation process. Because of their pressing needs and duties, informal caregivers are a challenging population to reach and involve in research [57]. However, the number of caregivers and experts that we included in our study was adequate for the qualitative methods used and is higher than the minimum recommended by the WHO guidelines to adapt iSupport to local contexts [22]. In addition, we set up a CAB that included both stakeholders and caregivers who worked continually and with great dedication through the adaptation process of iSupport. Second, the discrepancy in FG size between formal and informal caregivers and the attendance of informal caregivers in >1 FG may have contributed to reaching data saturation, but this may have reduced social desirability bias, thanks to both the progressive cementing of positive small group dynamics among participants and the variety of the contents discussed. Third, our study was conducted in Switzerland, a high-income country, equipped with a National Dementia Strategy that aims to improve the quality of life of people affected by dementia and to promote awareness and education on dementia [58]. Therefore, the feedback and experiences that we collected may not be easily generalized to all contexts. However, the adaptation strategies and phases described in our study may be useful for all countries interested in adapting digital interventions for caregivers of people with dementia, not only iSupport. Our findings suggest that digital interventions benefit from a community-based participatory approach and the involvement of caregivers to ensure that the final program meets the needs and preferences of users [17].

### Future Research

The recommendations and feedback that we collected during this study allowed us to adapt the original contents of the iSupport program to the Swiss context and to inform the development of the iSupport desktop version, mobile app, and printed manual. Following the Medical Research Council guidelines for the development of complex health interventions [59], we will proceed to assess the usability and feasibility of iSupport before its implementation. Evidence not only on the effectiveness but also on the ease of implementation and scalability of caregivers’ interventions is still rare in our country. We are determined to design and conduct good-quality studies to address these gaps and to promptly disseminate our findings and experience widely through peer-reviewed publications, the WHO knowledge exchange platform [60], and the global WHO iSupport network coordinated by the Brain Health Unit at the WHO.

Finally, the iSupport original program was developed by the WHO based on evidence related to carer training and support interventions and in collaboration with experts and caregivers [21]. Therefore, the program can be adapted to the extent that it maintains the original aims and structure [22]. During the study, we collected recommendations and feedback that would have required a consistent change in terms of resources and digital infrastructure to be implemented. These included, for instance, contents based on the type of dementia and stage of the disease, a comprehensive map of all digital and local resources available, and consultation from professionals as well as legal and financial assistance. Therefore, further development of iSupport could focus on supporting specific groups of caregivers, such as young carers or caregivers of people with rare dementia, and on providing personalized support tailored to the stage of the caregiver journey and the care needs of the care recipient.

### Conclusions

Despite the recognized importance of culturally adapting interventions to implement them in real-world settings, the evidence on how to conduct this process is still limited. Our study enriches this landscape by underscoring that an active engagement of the final users and stakeholders allows to adapt an intervention to their culture, values, and needs. In addition, this study provides examples of concrete strategies and methods to involve community members and stakeholders across different phases of the intervention. Indeed, despite the emerging importance of coconstructing research together with people as collaborators, rather than as simply *subjects* of traditional research, there is limited evidence regarding the modalities of this practice.

Our experience confirms that the adoption of a CBPR approach is necessary to identify and address criticisms and potential barriers to the use and acceptance of a digital educational intervention before its implementation. In conclusion, we envision this study as a potential driver for enhancing a more robust dialogue between researchers and communities. We firmly believe that CBPR represents a transformative research opportunity where the needs of academics and community members can be met and where both groups can find opportunity for mutual knowledge exchange and growth.

## Appendix

Multimedia Appendix 1 Community advisory board agreement.

Multimedia Appendix 2 Informed consent.

Multimedia Appendix 3 World Health Organization adaptation spreadsheet.

Multimedia Appendix 4 Summary of adaptations.

## Abbreviations

CAB: community advisory board
CBPR: community-based participatory research
FG: focus group
REDCap: Research Electronic Data Capture
WHO: World Health Organization

## Data Availability

The data sets generated and analyzed during this study are available from the corresponding author on reasonable request.
