# Supplementary material for: Culturally Adapting the World Health Organization Digital Intervention for Family Caregivers of People With Dementia (iSupport): Community-Based Participatory Approach
Source: JMIR Form Res. 2024 Jan 24;8:e46941. doi: 10.2196/46941 (PMC10851118; doi:10.2196/46941)
Supplement: Multimedia Appendix 1 [file formative_v8i1e46941_app1.pdf]

Università
della
Svizzera
italiana

Facoltà
di
scienze
biomediche

Via Buffi 13
6900 Lugano
Svizzera

Comitato Consultivo

Lettera d'intenti

Il presente accordo definisce la struttura, il ruolo e le responsabilità dei membri del comitato consultivo relativamente al progetto di ricerca iSupport.

Struttura

Un comitato consultivo è un organo composto da persone che condividono un'identità comune, un percorso storico, una lingua e/o una cultura d'interesse per lo sviluppo di un progetto di ricerca. I membri di un comitato consultivo partecipano volontariamente e attivamente alle diverse fasi del progetto, non solo nel ruolo di "consiglieri" ma di veri e propri esperti, sulla base dell'esperienza che li lega all'ambito della ricerca. Il comitato consultivo di iSupport prevede la partecipazione, oltre che dell'équipe di ricerca, di familiari di persone con demenza, operatori sanitari, rappresentanti di istituzioni pubbliche private che si occupano di demenza, sponsor e finanziatori del progetto.

Obiettivo

La scelta di istituire un comitato consultivo per iSupport è dettata dall'obiettivo principale di fornire uno strumento il più possibile vicino e rispondente alle esigenze della popolazione a cui è rivolto, riducendo il divario tra il mondo della ricerca e la comunità a cui questa è diretta.

Ruolo e responsabilità

Ai membri del comitato consultivo è chiesto di collaborare con l'équipe di ricerca fornendo pareri, suggerimenti e valutazioni sia sui contenuti di iSupport che sui risultati emersi lungo le diverse fasi della ricerca, con la possibilità di modificare in parte il progetto e sviluppare azioni future.

In particolare, i membri del Comitato Consultivo si impegnano a:

- Partecipare agli incontri - **circa 2 all'anno** - stabiliti dall'équipe di ricerca sulla base della disponibilità dei membri del comitato.

- Visionare il materiale inviato dall'équipe e svolgere le attività proposte – suddivise sulla base della disponibilità di ciascun membro-
- Dialogare e confrontarsi con l'équipe e gli altri membri del Comitato sugli aspetti emersi nel corso del progetto, sia durante gli incontri di gruppo che via mail.
- Mantenere la riservatezza rispetto ai contenuti emersi o dati sensibili con cui si entra in contatto.
- Rispettare il parere degli altri membri del Comitato.

L'équipe di ricerca si impegna a:

- Organizzare gli incontri e comunicarli tempestivamente via mail a tutti i membri.
- Proporre attività e inviare il materiale e le indicazioni necessarie tempestivamente.
- Aggiornare periodicamente, anche via mail, il comitato riguardo l'andamento del progetto e i risultati emersi.
- Mantenere la riservatezza rispetto ai contenuti emersi o dati sensibili con cui si entra in contatto.
- Accogliere e discutere suggerimenti o proposte di modifiche al progetto di ricerca.

Durata
Il presente accordo ha una validità stimata di tre anni.
La partecipazione al Comitato è volontaria e ogni membro ha diritto di uscire dal Comitato in qualsiasi fase della ricerca.

Lugano, 08/06/21

Equipe di ricerca

Nome e cognome:

Firma:

Membro del Comitato Consultivo

Nome e cognome:

Firma:
