# Supplementary material for: Culturally Adapting the World Health Organization Digital Intervention for Family Caregivers of People With Dementia (iSupport): Community-Based Participatory Approach
Source: JMIR Form Res. 2024 Jan 24;8:e46941. doi: 10.2196/46941 (PMC10851118; doi:10.2196/46941)
Supplement: Multimedia Appendix 2 [file formative_v8i1e46941_app2.pdf]

iSupport

Sviluppo di un sito web e di un’applicazione per smartphone e tablet per i familiari curanti di persone affette da demenza nel Canton Ticino

Lo studio è organizzato da

Università della Svizzera italiana (USI)

Dipartimento della sanità e della socialità del Canton Ticino (DSS)

Pro Senectute Svizzera

Scuola universitaria professionale della Svizzera italiana (SUPSI).

Gentile Signora/e,

Ci rivolgiamo a lei per chiederle se desidera partecipare ad un progetto di ricerca in campo della salute. Questo documento presenta il progetto di ricerca.

Spiegazione dettagliata dello studio

Premessa

Si stima che in Ticino circa il 50% delle persone affette da demenza risieda presso il proprio domicilio. I familiari curanti occupano dunque un ruolo centrale nella presa a carico e cura della persona affetta da demenza. Tale ruolo può rivelarsi difficile, stressante ed estenuante.

La strategia cantonale sulle demenze, tra i vari scopi, intende rafforzare e migliorare i servizi di cura e assistenza dei malati presso il domicilio. In particolare, è volta a potenziare il sostegno al familiare curante e a studiare ausili o supporti tecnologici utili a migliorare la qualità della presa in carico.

Il nostro progetto si allinea a tali obiettivi avvalendosi di iSupport, un programma di formazione online creato dall'Organizzazione Mondiale della Sanità (OMS) che aiuta i familiari curanti non solo a fornire una buona assistenza, ma anche a prendersi cura di sé stessi (WHO, 2019).

1. Scopo dello studio

In linea con la strategia cantonale sulle demenze, l'obiettivo principale di questo progetto è la valorizzazione della figura del familiare curante attraverso la formazione.

A tale scopo, intendiamo rendere il programma di iSupport pienamente fruibile alla popolazione ticinese, adattando e contestualizzando il suo contenuto alla realtà socio-culturale locale, e sviluppando un sito web e una app per smartphone e tablet. La

stretta collaborazione tra USI e SUPSI permetterà inoltre di sviluppare un modulo di formazione per gli operatori dei servizi socio-sanitario-assistenziali del Cantone.

2. Selezione dei partecipanti

I partecipanti saranno sia familiari curanti che operatori socio sanitari residenti nel canton Ticino e con esperienza nell'assistenza a persone affette da demenza.
Per familiare curante si intende si intende una persona che presta regolarmente assistenza, sorveglianza e accompagnamento, a titolo non professionale e in maniera totale o parziale, a una persona dipendente da terzi. Nella maggior parte dei casi questa figura ha un vincolo di parentela con la persona assistita. Per operatori socio-sanitari si intendono persone che prestano assistenza professionale all'interno di strutture di cura o a domicilio ad utenti non autonomi o parzialmente autonomi.
I partecipanti verranno reclutati principalmente tramite i partner del progetto (Pro Senectute, Alzheimer Ticino e Centro Competenze Anziani SUPSI) e stratificati per genere, età, esperienza nella cura, educazione ed eventuale relazione familiare con la persona con demenza. Unico criterio di esclusione è l'impossibilità di avere un accesso regolare a un computer con Internet.

3. Informazioni generali sullo studio

Lo studio mira a coinvolgere i partecipanti nella creazione di un programma online di supporto a chi presta assistenza a titolo formale o informale a una persona con demenza. La raccolta dei bisogni, opinioni e abitudini dei partecipanti facilita la costruzione di uno strumento il più rispondente possibile alle reali esigenze dei familiari curanti a cui iSupport è rivolto.

4. Svolgimento dello studio

Lo studio prevede diverse tappe, tra cui si possono distinguere tre fasi principali: una fase di adattamento linguistico e culturale di iSupport al contesto ticinese, una fase pilota di implementazione del programma e una fase di realizzazione e monitoraggio di app e sito.

Nella prima fase, i partecipanti vengono invitati tramite focus group condotti da un facilitatore, a valutare la comprensibilità e l'adeguatezza culturale dei singoli moduli, già tradotti in italiano.
All'interno del focus group, i partecipanti vengono a invitati a raccontare la propria esperienza nell'assistenza, a valutare e commentare i contenuti presentati di iSupport e a impegnarsi in attività pratiche e interattive, con la possibilità di fornire spunti e modifiche al programma.
Il focus group a cui lei è invitato si svolgerà in presenza presso l'Università della Svizzera Italiana a Lugano ed è composto da altri familiari curanti, da un facilitatore che orienterà la discussione, da un osservatore che prenderà nota dei contenuti emersi e da uno o più membri dell'équipe di ricerca. La durata della discussione è di circa 120 minuti e verrà audioregistrata per permettere successivamente di analizzare i contenuti emersi.
La sua adesione ai focus group le permetterà, se interessato, di entrare in contatto con il gruppo di ricerca, che si impegna a mantenerla informata sui risultati della ricerca e lo sviluppo del progetto.

5. Benefici

La sua partecipazione a questo studio contribuirebbe alla creazione di uno strumento di formazione che, una volta implementato, potrà sostenere e aiutare nell'assistenza gli operatori socio-sanitari e i familiari curanti del Canton Ticino. Inoltre, la sua partecipazione le permette di venire in anteprima a conoscenza dei contenuti del manuale di iSupport.

6. Diritti

La sua partecipazione a questo studio è volontaria. Inoltre, è possibile ritirarsi dallo studio in qualsiasi momento, senza dover giustificare il motivo.Tutte le informazioni raccolte da noi sono interamente confidenziali.

7. Obblighi

Se decide di partecipare, la preghiamo di seguire le seguenti indicazioni:

- Parlare uno alla volta per dare la possibilità a tutti di esprimere il proprio parere

- Rispettare le opinioni altrui, anche in caso di disaccordo
- Dare il parere più onesto e sincero possibile: non ci sono risposte giuste o sbagliate
- Rispettare la riservatezza dei contenuti emersi nel gruppo

8. Rischi

La partecipazione a questo studio non la espone ad alcun rischio fisico.
Potrebbe provare qualche disagio a dare il suo parere all'interno di un gruppo, le ricordiamo che non è obbligato/a a esporsi, che i dati emersi all'interno del gruppo sono coperti da privacy e che ogni partecipante è invitato alla riservatezza dei contenuti emersi durante i focus group.

9. Risultati

Durante lo studio, il ricercatore le comunicherà qualsiasi nuovo sviluppo che potrebbe avere ripercussioni sui benefici dello studio o sulla sua sicurezza e, di conseguenza, influire sul suo consenso alla partecipazione.

10. Confidenzialità dei dati personali

Nell'ambito di questo studio raccoglieremo alcuni suoi dati personali e registreremo con il suo consenso i contenuti emersi all'interno dei focus group.
Rispettiamo tutte le disposizioni legislative in materia di protezione dei dati. Tutti i dati sono raccolti e conservati in forma codificata e protetta. I dati vengono codificati in fase di rilevazione. Codificare significa che tutti i dati che potrebbero identificare il partecipante (nome, data di nascita) vengono cancellati e sostituiti da un codice, che impedisce a chiunque non lo conosca di risalire al partecipante. L'elenco dei codici di decodifica rimane sempre all'interno dell'istituzione.
E' possibile che alcuni commenti o riflessioni emerse durante il focus group possano essere riutilizzati all'interno di riviste e articoli medici scientifici; anche in questo caso i dati individuali sono sempre codificati e non è possibile risalire ai partecipanti. Tutte le persone con accesso ai dati del progetto sono tenute al rispetto del segreto professionale.

11. Ritiro dallo studio

Se lo desidera, può ritirarsi dallo studio in qualsiasi momento. I dati rilevati fino a quel momento saranno comunque valutati. Dopo l'analisi i suoi dati verranno completamente anonimizzati. Ciò significa che il codice di decodifica verrà distrutto in modo tale che nessuno avrà modo di risalire alla provenienza dei dati.

12. Indennità per i partecipanti allo studio

Non è prevista alcuna indennità o compenso. La partecipazione allo studio non causerà costi né a lei né alla sua assicurazione malattia.

13. Responsabilità

La responsabilità civile dell'Università della Svizzera italiana risponderà se, in conseguenza dello studio, dovesse subire un danno.

14. Finanziamento dello studio

Lo studio è finanziato dal Dipartimento della sanità e della socialità del Canton Ticino (DSS), dall'Università della Svizzera italiana e da Pro Senectute Svizzera.

15. Contatti di riferimento

In caso di dubbi, timori o emergenze che dovessero insorgere durante o dopo lo studio, può rivolgersi in ogni momento alle persone di contatto indicate di seguito:

Dr. Maddalena Fiordelli e Anna Messina
Facoltà di scienze biomediche,
Istituto di Salute Pubblica
Università della Svizzera italiana (USI)
Via Buffi 13, 6900 Lugano
+ 41 (0) 58 666 4130
isupport@usi.ch

Dichiarazione di consenso

Legga attentamente il presente formulario. Non esiti a porre domande se qualcosa non le è chiaro o se desidera una spiegazione.

**Numero BASEC dello studio:** 2020-02030 / CE 3731
**Istituzione responsabile:** Università della Svizzera italiana
**Responsabile dello studio nel luogo dello studio:** Prof. Emiliano Albanese (USI)
**Luogo dello studio:** Ticino

**Dati del partecipante**
Cognome
Nome
Data di nascita
Donna / Uomo

- Sono stato/a informato/a dallo/a sperimentatore/rice sottoscritto/a oralmente e per scritto in merito allo scopo, allo svolgimento dello studio, agli svantaggi e ai vantaggi nonché agli eventuali rischi.
- Partecipo allo studio volontariamente e accetto il contenuto del documento informativo scritto fornito in relazione allo studio sopra menzionato.
- Ho avuto tempo a sufficienza per prendere la mia decisione.
- Ho ricevuto risposte esaustive alle mie domande relative alla partecipazione a questo studio.
- Posso conservare il documento informativo scritto sullo studio e ricevo una copia della mia dichiarazione di consenso scritta.
- Acconsento che i contenuti espressi durante lo studio siano registrati e trascritti in osservanza dei rispetto della privacy
- Acconsento al fatto che gli specialisti competenti del committente dello studio, del comitato etico competente per questo studio possano accedere ai miei dati

originali a fini di esame e controllo, tuttavia in stretta osservanza della confidenzialità.

- So che i miei dati personali potranno essere trasmessi solo in forma codificata a scopo di ricerca per questo progetto di ricerca.
- Posso revocare il mio consenso in ogni momento e senza addurre alcuna motivazione. Sono d'accordo che i miei dati rilevati fino a quel momento siano comunque valutati.
- La responsabilità civile dell'istituzione risponderà di eventuali danni.
- Sono consapevole della necessità di rispettare gli obblighi menzionati nel documento informativo durante lo studio.

Luogo e data	Firma del/ della partecipante o del rappresentante legale (per adulti sotto tutela)
______________________________	____________________
