# Supplementary material for: Culturally Adapting the World Health Organization Digital Intervention for Family Caregivers of People With Dementia (iSupport): Community-Based Participatory Approach
Source: JMIR Form Res. 2024 Jan 24;8:e46941. doi: 10.2196/46941 (PMC10851118; doi:10.2196/46941)
Supplement: Multimedia Appendix 4 [file formative_v8i1e46941_app4.docx]

| Dimension and issues  **Multimedia appendix 4**. Summary of adaptations. | | **Original text** | **Proposed adaptation (Italian versions are given in italics)** |
| --- | --- | --- | --- |
| Language | | | |
|  | **Familiarity: some original expressions did not sound familiar enough or were not frequently used in the local context** | | |
|  |  | “Informal carer” | “Family carer” (*familiare curante*) |
|  |  | “Community center” | “Day care centre” (*centro diurno*) |
|  |  | “Rural areas” | “Remote areas” (*zone isolate*) |
|  |  | “Paid in-home helper” | “Domestic worker” (*assistente familiare*) |
|  |  | “Drugs” | “Medications” (*medicamenti*) |
|  | **Sensitivity: some original expressions used to designate the person who lived with dementia or the carer were considered stigmatizing or not sensitive enough** | | |
|  |  | “Person with dementia”; “person who suffers from dementia” | “Person affected by dementia” (*persona affetta da demenza*); “person assisted by” (*persona assistita*) |
|  |  | “It’s normal to feel frustrated” | “It’s comprehensible to feel frustrated” (*è comprensibile sentirsi frustrati*) |
|  |  | “The person you care for” | “The person you take care of” (*la persona di cui ti prendi cura*); “the person you assist” (*la persona che assisti*) |
|  |  | “Show compassion” | “Show comprehension” (*mostrare comprensione*) |
|  | **Precision: some expressions were not considered accurate enough or correct** | | |
|  |  | “Alzheimer’s disease is the most common cause of dementia” | “Alzheimer’s disease is the most common type of dementia” (*la malattia di Alzheimer è una delle forme di demenza più diffuse*) |
|  |  | “Memory loss” | “Memory impairment” (*difficoltà di memoria*) |
|  |  | “To feel full” | “To feel satiated” (*sentirsi sazi*) |
|  |  | “Things that can or cannot be eaten” | “Edible or inedible substances” (*sostanze commestibili o non commestibili*) |
|  |  | “Helpful/ or unhelpful thoughts” | “Functional or dysfunctional thoughts” (*pensieri funzionali e disfunzionali*) |
|  |  | “Getting confused about the time” | “Feel disoriented” (*sentirsi disorientato nel tempo e nello spazio*) |
|  |  | “Doing things over and over” | “Repetitive behaviour” (*comportamento ripetitivo*) |
|  |  | “Changes in the brain” | “Cognitive impairment” (*decadimento cognitivo*) |
|  | **Learning approach: current terms that were reminiscent of an overly scholastic and educational approach were replaced with more general expressions** | | |
|  |  | “Lesson” | “Chapter” (*capitolo*) |
|  |  | “Learn” | “Know more about dementia” (*conoscere di più la demenza*) |
|  |  | “Teach skills” | “This chapter will help you in preventing and coping with behavior changes” (*questo capitolo ti aiuterà a prevenire e gestire i cambiamenti nel comportamento della persona che assisti*) |
|  | **Use of English: English expressions that are also used in spoken Italian were replaced with Italian terms** | | |
|  |  | “Focus of the manual” | *Obiettivo del manuale* |
|  |  | “Relax” | *Rilassa* |
|  |  | “Status” | *Condizione* |
|  |  | “Stress” | *Preoccupazione* |
|  | **Use of numbers: percentages were transformed from a numerical to an alphabetical representation** | | |
|  |  | “20%-30%” | Approximately one-third (*circa un terzo*) |
|  | **Informal language: some expressions were found to be “childish” or too informal** | | |
|  |  | “You finished the lesson, well done” | “You finished the chapter, let’s go to the next!” (*hai completato il capitolo, passa al successivo!*) |
|  |  | “Take a nap” | “Rest” (*riposa*) |
| Resources | | | |
|  | **Need to specify local resources for dementia; need to specify local resources for mental health** | | |
|  |  | N/A^a^ | The names and contacts of several local organizations and Alzheimer associations were specified in different parts of the program and in the last chapter; link to psychological support resources was added in M3^b^ and M5^b^ |
| Contents | | | |
|  | **Need to adapt leisure activities to local culture** | | |
|  |  | N/A | Local leisure activities added: take a trip, enroll in a course, visit museums, go to the movies or theater, and go out for dinner |
|  | **Need to adapt names to local cultures** | | |
|  |  | Olivia has dementia and lives with her husband Jacob | *Anna è affetta da demenza e vive con suo marito Marco* |
|  | **Case scenarios: some answer options were found offensive for the caregiver or unfamiliar** | | |
|  |  | Exercise (M5.09^c^): “What would you recommend to Mateo’s family?” An answer option was “Shout at Mateo, shaming him for his conduct” | The answer option was deleted because it sounded offensive to the carer |
|  |  | Exercise (M5.04^c^): “How do you think Sofia should react?” An answer option was “Sofia could for example read the newspaper to him, cook his favorite dish or visit a shop to let him straighten the shelves with the shopkeeper’s permission” | The text “visiting a shop to let him straighten the shelves with the shopkeeper’s permission” was removed because it was found unfamiliar |
|  | **The description of case scenarios or answer options often did not represent the variety and differences of caregiving experiences** | | |
|  |  | Disclaimer added | “Remember that there are not always one-size-fits-all solutions to a problem; there may be others more suited to your personal situation” (*Ricorda che non sempre esistono soluzioni uniche a un problema, potrebbero essercene altre più adatte alla tua situazione personale*) |
| Graphics | | | |
|  | **Cartoon illustrations were often found childish, representing inanimate objects, and not consistent with the contents** | | |
|  |  | Cartoon illustrations | Cartoon illustrations were replaced with human illustrations, representing the variety of caregiving experiences and divided per module |
| Added features | | | |
|  | Glossary | | A glossary with the main recurrent terms used throughout the program and their explanations was added to the user’s guide |
|  | Interactive forum | | A forum section was included at the end of each chapter where participants can interact by writing with other users |
|  | Read-aloud option | | A read-aloud option was added to the relaxation exercises |
|  | Navigation survey | | An initial short questionnaire was added to personalize the navigation of the user based on their situation and need |
